# Effectiveness of 23-valent pneumococcal polysaccharide vaccination in preventing community-acquired pneumonia hospitalization and severe outcomes in the elderly in Spain

**DOI:** 10.1371/journal.pone.0171943

**Published:** 2017-02-10

**Authors:** Àngela Domínguez, Núria Soldevila, Diana Toledo, Núria Torner, Luis Force, María José Pérez, Vicente Martín, Lourdes Rodríguez-Rojas, Jenaro Astray, Mikel Egurrola, Francisco Sanz, Jesús Castilla, Working Group of the Project PI12/02079

**Affiliations:** 1 Departament de Salut Pública, Universitat de Barcelona, Barcelona, Spain; 2 CIBER Epidemiología y Salud Pública (CIBERESP), Madrid, Spain; 3 Agència de Salut Pública de Catalunya, Barcelona, Spain; 4 Hospital de Mataró, Mataró, Spain; 5 Hospital Universitario Virgen de Valme, Sevilla, Spain; 6 Universidad de León, León, Spain; 7 Hospital Ramón y Cajal, Madrid, Spain; 8 Consejería de Sanidad, Madrid, Spain; 9 Hospital de Galdakao, Usansolo, Spain; 10 Consorci Hospital General Universitari de Valencia, Valencia, Spain; 11 Instituto de Salud Pública de Navarra, IdiSNA, Pamplona, Spain; Public Health England, UNITED KINGDOM

## Abstract

Pneumococcal pneumonia is a serious cause of morbidity and mortality in the elderly, but investigation of the etiological agent of community-acquired pneumonia (CAP) is not possible in most hospitalized patients. The aim of this study was to estimate the effect of pneumococcal polysaccharide vaccination (PPSV23) in preventing CAP hospitalization and reducing the risk of intensive care unit admission (ICU) and fatal outcomes in hospitalized people aged ≥65 years. We made a multicenter case-control study in 20 Spanish hospitals during 2013–2014 and 2014–2015. We selected patients aged ≥65 years hospitalized with a diagnosis of pneumonia and controls matched by sex, age and date of hospitalization. Multivariate analysis was performed using conditional logistic regression to estimate vaccine effectiveness and unconditional logistic regression to evaluate the reduction in the risk of severe and fatal outcomes. 1895 cases and 1895 controls were included; 13.7% of cases and 14.4% of controls had received PPSV23 in the last five years. The effectiveness of PPSV23 in preventing CAP hospitalization was 15.2% (95% CI -3.1–30.3). The benefit of PPSV23 in avoiding ICU admission or death was 28.1% (95% CI -14.3–56.9) in all patients, 30.9% (95% CI -32.2–67.4) in immunocompetent patients and 26.9% (95% CI -38.6–64.8) in immunocompromised patients. In conclusion, PPSV23 showed a modest trend to avoidance of hospitalizations due to CAP and to the prevention of death or ICU admission in elderly patients hospitalized with a diagnosis of CAP.

## Introduction

*Streptococcus pneumoniae* is a leading cause of serious illness, along with bacteremia, meningitis and pneumonia. In adults aged ≥65 years, most invasive cases result from the complications of pneumonia [1]. Pneumococcal disease causes a substantial burden among older adults [2], and up to one third of patients require intensive care unit (ICU) admission and nearly 20% die during hospitalization or in the first month after discharge [3].

Recognition of continued morbidity and mortality due to pneumococcal infections despite the use of appropriate antibiotics led to increased interest in disease prevention by vaccination and, since 1983, a 23-valent pneumococcal polysaccharide vaccine (PPSV23) containing antigens against 23 of the 94 serotypes has been available [4]. Post-licensure studies showed the vaccine is protective against invasive disease in immunocompetent older adults [5–7]. Evidence in support of a beneficial effect of PPSV23 in preventing pneumococcal pneumonia is more limited.

Until the 13-valent conjugate pneumococcal vaccination (PCV13) for adults recently became available, PPSV23 vaccination was recommended in the United States for all persons aged ≥65 years and for adults aged <65 years at increased risk of invasive pneumococcal disease [8]. Similar recommendations are in place in most European Union countries, although in France, the Netherlands, and Sweden vaccination is recommended only for adults with high risk conditions and not for healthy adults aged ≥65 years [9]. Currently, the recommended pneumococcal vaccination schedule for adults aged ≥65 years in the United States and other countries is the administration of PCV13 followed by PPSV23 at least 1 year after PCV13 if the subject has not received any previous pneumococcal vaccine and a dose of PCV13 if they have previously received a PPSV23 dose (1 year after PPSV23) [10,11]. In adults aged ≥65 years with immunocompromising conditions, functional or anatomic asplenia, cerebrospinal fluid leaks or cochlear implants, the recommended interval between PCV13 followed by PPSV23 is ≥8 weeks [12]. In contrast, in Spain as in other countries, the current recommendation is to maintain PPSV23 vaccination in persons aged ≥ 65 years [13,14]. By the end of 2016, all Spanish regions included the PCV13 vaccine in the pediatric vaccination calendar; previously, only two regions had included it.

Because samples for the investigation of the etiological agent are not always collected, pathogens are detected in only one third of all cases of community-acquired pneumonia (CAP) [15] and, due to limitations in the sensitivity and specificity of the available diagnostic test, the cause of pneumonia cannot be identified in most hospitalized patients [16]. Therefore, CAP is an outcome of public health relevance that does not depend on the etiologic diagnosis, and thus the study of PPSV23 vaccine effectiveness (VE) against hospitalized cases of CAP may be considered a proxy of VE against pneumococcal pneumonia.

In Spain, CAP remains a major health problem in older adults [17] and free, universal PPSV23 vaccination of people aged ≥65 years has progressively been included in the vaccination schedule of some regions from 1999 onwards [18], although the coverage at any time before data collection has remained < 50% [19]. According to the standing order strategy [20], PPSV23 is administered in primary care centers or hospitals without requiring a specific order from the attending physician [21].

Because *S*. *pneumoniae* infection in the elderly may result not only in CAP but also death [22], it may be of interest to assess the benefit of pneumococcal vaccination in protecting against the worst CAP outcomes, such as ICU admission and death.

The objective of this study was to estimate the effect of pneumococcal polysaccharide vaccination in preventing CAP hospitalization in hospitalized subjects aged ≥65 years and reducing the risk of severe and fatal outcomes in CAP hospitalized subjects aged ≥65 years.

## Methods

### Study design

We carried out a multicenter case-control study in 20 hospitals from seven Spanish regions (Andalusia, Castile and Leon, Catalonia, Madrid, Navarra, the Basque Country and Valencia Community). Cases and corresponding controls admitted to participating hospitals between September 2013 and June 2015 were recruited.

### Selection of cases and controls

We selected patients aged ≥65 years hospitalized for at least 24h with a diagnosis of CAP. The diagnosis of pneumonia was based on the finding of a new infiltrate typical of pneumonia on chest radiography, fever and any symptoms of lower respiratory tract infection. Pneumonia was considered as nosocomial, and therefore excluded, if the onset of symptoms occurred more than 48h after hospital admission [23].

One matched control was selected for each case from among patients with unplanned hospital admission due to causes other than pneumonia or acute respiratory disease. Controls were matched according to sex, age (±3 years) and date of hospitalization (preferentially ±10 days but ±30 days if no appropriate control was found using the ±10 day interval) and were selected from patients admitted to the internal medicine service, general surgery, otorhinolaryngology, ophthalmology, dermatology, or traumatology services. When there was more than one possible control for a case, the patient with a date of hospitalization ±10 days and with the age closest to the case was chosen. Patients referred from nursing homes and those who did not provide written informed consent were excluded.

### Data collection

The following demographic variables and pre-existing medical conditions were recorded: age, sex, marital status, educational level, smoking status, high alcohol consumption (>40gr/day for men and >24gr/day for women), number of hospital visits during the last year, whether the patient lived alone or with cohabitants, the Barthel index as a measurement of limitations in activity (ranging from 0 -complete dependence- to 100 -complete independence), chronic obstructive pulmonary disease (COPD), chronic respiratory failure, other lung diseases, neoplasia, transplantation, immunosuppressive treatment, asplenia, diabetes, renal failure, nephrotic syndrome, autoimmune disease, AIDS, HIV infection, congestive heart disease, disabling neurological disease, chronic liver disease, hemoglobinopathy or anemia, and cognitive dysfunction. A severe outcome was defined as ICU admission or death. Information on influenza vaccination in the current season and pneumococcal vaccination was collected by review of the hospital medical record and, if this information was not contained in the hospital medical record, the primary care medical.

Given that antibody concentrations and effectiveness of the vaccine decline after 5–10 years in elderly persons [24,25], the main analysis was made considering as vaccinated with the pneumococcal vaccine cases and controls who had received a dose of PPVS23 ≥14 days and in the 5 years before symptom onset (cases) or before symptom onset of the matched case (controls). All other subjects were considered unvaccinated.

Cases were considered vaccinated with the current seasonal influenza vaccine if they had received a dose of the vaccine ≥14 days before symptom onset. Controls were considered vaccinated if they had received a dose of the vaccine at least 14 days before the onset of symptoms of the matched case.

### Sample size calculation

The minimum sample size required, calculated using Schlesselman’s criteria [26], assuming a PPSV23 rate among controls of 27.5%, a vaccination effectiveness of 24%, a statistical power of 80% and a confidence level of 95% according to previous studies [27], was 1118 cases and 1118 controls.

### Statistical analysis

A bivariate comparison for matched data of demographic variables and medical conditions between cases and controls was made using McNemar’s test. A two-tailed distribution was assumed for all p-values.

To control for the possible influence of influenza viruses on CAP hospitalization, we considered two periods in each season: an epidemic period including the weeks when influenza viruses circulated in Spain and a non-epidemic period including the remaining weeks. According to the reports of the Spanish network for epidemiological surveillance [28,29], epidemic weeks were 25 November to 20 April in the 2013–2014 season and 24 November to 19 April in the 2014–2015 season.

The interaction between PPSV23 and the other variables was analyzed.

Vaccine effectiveness (VE) was calculated using the formula: VE = (1 –OR) x 100.

A univariate conditional logistic regression model was used to estimate the crude VE in preventing CAP hospitalization. Propensity score (PS) analysis was used to evaluate the adjusted vaccine effectiveness. The PS was created using a logistic regression model with PPSV23 vaccination status as the outcome and demographic variables, Barthel index, smoking and alcohol intake, number of hospital visits, comorbidities, epidemic period and influenza vaccination as independent variables. The PS was used as a covariate in the final conditional logistic regression model.

To assess the benefit of PPSV23 in avoiding severe outcomes in hospitalized patients we compared the characteristics of hospitalized patients with CAP who died or were admitted to the ICU with those of other hospitalized patients with CAP using unconditional logistic regression. We created a PS using a logistic regression model with PPSV23 vaccination status as the outcome and demographic variables, Barthel index, smoking and alcohol intake, number of hospital visits, comorbidities, epidemic period and influenza vaccination as independent variables. The PS was used as a covariate in the final unconditional logistic regression model.

The analysis was performed using the SPSS v.23 statistical package and the R v3.3.0 statistical software (http://cran.r-project.org).

### Ethical considerations

All data collected were treated as confidential, in strict observance of legislation on observational studies. The study was approved by the Ethics Committees of the participating hospitals (Comité Ético de Investigación Clínica del Hospital Clínic de Barcelona; Comité Ético de Investigación Clínica del Hospital Universitari Mutua de Terrassa; Comité Ético de Investigación Clínica de la Corporació Sanitaria Parc Taulí de Sabadell; Comité Ético de Investigación Clínica del Hospital de Mataró, Consorci Sanitari del Maresme; Comité Ètic d’Investigació Clínica de la Fundació Unio Catalana Hospitals; Comité Ético de Investigación Clínica Área de Euskadi; Comité Ético de Investigación Clínica Área de Salud de Burgos y Soria; Comité Ético de Investigación Clínica Área de Salud de León; Comité Ético de Investigación Clínica Área de Salud Valladolid-Este; Comité Coordinador de Ética de la Investigación Biomédica de Andalucía; Comité Ético de Investigación Clínica del Hospital Ramón y Cajal, Madrid and Comité Ético de Investigación Clínica del Consorcio Hospital General Universitario de Valencia). Written informed consent was obtained from all patients included in the study.

## Results

A total of 1895 cases and 1895 controls were included in the study. The distribution of cases and controls according to demographic variables, medical conditions and vaccination history is shown in Table 1.

**Table 1:** Distribution of cases and controls according to demographic variables, medical conditions and vaccination history.

Characteristics	Cases (N = 1895)	Controls (N = 1895)	Crude OR (95% CI)	p-value
**Age group**				
65–74 years	592 (31.2%)	603 (31.8%)	1	
75–84 years	879 (46.4%)	897 (47.3%)	1.17 (0.84–1.64)	0.36
≥85 years	424 (22.4%)	395 (20.8%)	1.69 (1.07–2.68)	0.03
**Sex**				
Female	746 (39.4%)	746 (39.4%)	-	
Male	1149 (60.6%)	1149 (60.6%)	-	
**Marital status**				
Married/Cohabiting	1099 (58.0%)	1108 (58.7%)	1	
Single	140 (7.4%)	147 (7.8%)	0.96 (0.75–1.24)	0.77
Widowed	611 (32.3%)	607 (32.1%)	1.02 (0.87–1.20)	0.82
Separated/Divorced	44 (2.3%)	27 (1.4%)	1.66 (1.01–2.70)	0.04
**Educational level**				
Without or primary	1378 (73.5%)	1308 (70.3%)	1	
Secondary or higher	498 (26.5%)	553 (29.7%)	0.81 (0.69–0.95)	0.01
**Household size**				
Live alone	339 (17.9%)	363 (19.2%)	1	
Live with cohabitant	1555 (82.1%)	1526 (80.8%)	1.09 (0.93–1.29)	0.29
**Barthel index**				
0–90	766 (40.4%)	773 (40.8%)	0.98 (0.85–1.13)	0.80
>90	1129 (59.6%)	1122 (59.2%)	1	
**Smoking status**				
Non smoker	838 (44.2%)	983 (51.9%)	1	
Smoker	165 (8.7%)	145 (7.7%)	1.73 (1.31–2.27)	<0.01
Ex-smoker	892 (47.1%)	767 (40.5%)	1.78 (1.48–2.14)	<0.01
**High alcohol consumption**				
Yes	71 (3.7%)	54 (2.8%)	1.34 (0.93–1.93)	0.12
No	1824 (96.3%)	1841 (97.2%)	1	
**No. of hospital visits**				
0–2	922 (48.9%)	897 (47.8%)	1	
≥3	962 (51.1%)	978 (52.2%)	0.95 (0.82–1.10)	0.50
**Risk medical conditions**				
No	193 (10.2%)	255 (13.5%)	1	
Yes	1702 (89.8%)	1640 (86.5%)	1.38 (1.13–1.69)	0.002
**Epidemiologic week**				
Yes	1302 (68.7%)	1266 (66.8%)	1.92 (1.31–2.83)	0.001
No	593 (31.3%)	629 (33.2%)	1	
**Influenza vaccine**				
Yes	891 (47.0%)	855 (45.1%)	1.10 (0.95–1.27)	0.19
No	1004 (53.0%)	1040 (54.9%)	1	
**Pneumococcal polysaccharide vaccine in 5 previous years**				
Yes	259 (13.7%)	272 (14.4%)	0.94 (0.78–1.14)	0.53
No	1636 (86.3%)	1623 (85.6%)	1	

A total of 1003 cases (52.0%) and 923 controls (47.8%) had received pneumococcal vaccination and most (973 cases and 896 controls) had received PPSV23; only 5 cases and 5 controls had received PCV13 alone and 16 cases and 8 controls had received both vaccines; 259 cases and 272 controls had received PPSV23 in the previous 5 years. All patients who had received PCV13 were excluded from the study of VE.

Of the 1895 cases with CAP, the etiological agent was determined in 469 (24.7%) and, of these, *S*. *pneumoniae* was detected in 324 (69.1%).

Most patients hospitalized due to CAP (89.8%) and controls (86.5%) presented one or more comorbidities, whose distribution is shown in Table 2.

**Table 2:** Distribution of cases and controls according to comorbidities.

Characteristics	Cases (N = 1895)	Controls (N = 1895)	Crude OR (95% CI)	p-value
**Immunocompetent**				
Chronic respiratory failure	381 (20.1%)	210 (11.1%)	2.22 (1.82–2.71)	<0.001
Diabetes with complications	112 (5.9%)	134 (7.1%)	0.83 (0.64–1.07)	0.15
Diabetes without complications	534 (28.2%)	554 (29.2%)	0.95 (0.82–1.09)	0.47
Renal failure without hemodialysis	344 (18.2%)	369 (19.5%)	0.91 (0.77–1.08)	0.28
Autoimmune disease	89 (4.7%)	89 (4.7%)	1.00 (0.73–1.37)	1.00
Chronic obstructive pulmonary disease	570 (30.1%)	289 (15.3%)	2.60 (2.18–3.09)	<0.001
Congestive heart disease	528 (27.9%)	563 (29.7%)	0.90 (0.77–1.05)	0.17
Neurological disease	155 (8.2%)	121 (6.4%)	1.30 (1.02–1.66)	0.04
Chronic liver disease	72 (3.8%)	106 (5.6%)	0.66 (0.48–0.90)	0.01
Cognitive dysfunction	229 (12.1%)	220 (11.6%)	1.05 (0.85–1.29)	0.63
**Immunocompromised**				
Solid organ neoplasia	330 (17.4%)	398 (21.0%)	0.79 (0.67–0.93)	0.01
Hematologic neoplasia	47 (2.5%)	45 (2.4%)	1.04 (0.69–1.58)	0.83
Transplantation	20 (1.1%)	13 (0.7%)	1.54 (0.76–3.09)	0.23
Immunosuppressive treatment	73 (3.9%)	88 (4.6%)	0.82 (0.59–1.13)	0.22
Oral corticosteroid therapy	100 (5.3%)	61 (3.2%)	1.71 (1.22–2.38)	0.002
Asplenia	5 (0.3%)	4 (0.2%)	1.25 (0.34–4.65)	0.74
Renal failure with hemodialysis	31 (1.6%)	41 (2.2%)	0.75 (0.47–1.20)	0.23
Nephrotic syndrome	25 (1.3%)	9 (0.5%)	3.00 (1.35–6.68)	0.01
AIDS	3 (0.2%)	1 (0.1%)	3.00 (0.31–28.84)	0.34
HIV infection	3 (0.2%)	2 (0.1%)	1.50 (0.25–8.98)	0.66
Hemoglobinopathy or anemia	299 (15.8%)	328 (17.3%)	0.89 (0.74–1.06)	0.19

Of the 1895 cases, 130 died within 30 days of admission and 81 were admitted to the ICU, of whom 14 died.

No interaction between PPSV23 and comorbidities (p = 0.32) or age (p = 0.24) was observed32).

The adjusted effectiveness of PPSV23 against CAP hospitalization is shown in Table 3 and the benefit of PPSV23 in avoiding ICU admission or death in cases is shown in Table 4. The effectiveness of PPSV23 in preventing CAP hospitalization was 15.2% (95% CI -3.1–30.3) in all cases, which was not significant. The effectiveness of PPSV23 in preventing severe outcomes in cases was 28.1% (95% CI -14.3–56.9) in all patients, 30.9% (95% CI -32.2–67.4) in immunocompetent patients and 26.9% (95% CI -38.6–64.8) in immunocompromised patients.

**Table 3:** Crude and adjusted effectiveness of PPSV23 against hospitalization due to community-acquired pneumonia.

	Cases vaccinated^a^/N (%)	Controls vaccinated^a^/N (%)	Crude vaccine effectiveness(95% CI)	p-value	Adjusted vaccine effectiveness(95% CI)	p-value
**All**	259/1895 (13.7%)	272/1895 (14.4%)	5.8% (-13.7–21.9)	0.53	15.2% (-3.1–30.3)	0.10^b^
**65–74 years**	116/592 (19.6%)	133/592 (22.5%)	16.2% (-11.3–36.9)	0.22	23.6% (-3.0–43.3)	0.08^c^
**75–84 years**	94/879 (10.7%)	95/879 (10.8%)	1.2% (-34.5–27.5%)	0.94	11.5% (-22.1–35.9)	0.46^d^
**≥85 years**	49/424 (11.6%)	44/424 (10.4%)	-12.8% (-73.6–26.7)	0.58	0.3% (-57.7–36.9)	0.99^e^

^a^ In the 5 previous years. Adjusted for the propensity score.

Statistical power:

^b^ 41%,

^c^46%,

^d^12%,

^e^3%

**Table 4:** Effectiveness of the PPSV23 in avoiding intensive care unit admission or death in hospitalized patients with community-acquired pneumonia.

	Severe outcomes vaccinated^a^/N (%)	Non severe outcomes vaccinated^a^/N (%)	Crude vaccine effectiveness (95% CI)	p-value	Adjusted vaccine effectiveness (95% CI)	p-value
All	21/211 (10.0%)	238/1684 (14.1%)	32.9% (-5.2–59.2)	0.09	28.1% (-14.3–56.9)	0.18^b^
Immunocompetent	10/97 (10.3%)	147/974 (15.1%)	35.3% (-21.6–69.1)	0.21	30.9% (-32.2–67.4)	0.30^c^
Immunocompromised	11/114 (9.6%)	91/710 (12.8%)	27.4% (-35.0–64.4)	0.34	26.9% (-38.6–64.8)	0.36^d^

^a^ In the 5 previous years. Adjusted for the propensity score.

Statistical power:

^b^29%,

^c^20%,

^d^15%

S1 and S2 Tables show the VE excluding cases and controls vaccinated more than 5 years previously. The VE against hospitalization was lower (6.1%; -21.8 to 27.6), but was significantly higher (40.9%; 2.9–65.6; p = 0.04) in preventing ICU admission or death.

## Discussion

The results of this study show that PPSV23 vaccination resulted in a non-significant trend to protection against hospitalization due to CAP in the elderly and in preventing severe outcomes when persons vaccinated ≥5 years previously were considered unvaccinated, but offered significant protection against severe outcomes when cases and controls vaccinated ≥ 5 years previously were excluded from the analysis (VE: 40.9%; 2.9–65.6).

Comparison of our results with other studies of CAP hospitalization in the elderly that considered patients vaccinated if they had received PPSV23 in the previous 5 years shows some similarities. A case-control study in Japan in people aged ≥65 years found no association between vaccination in the previous 5 years and CAP [30]. In a Spanish cohort study in individuals aged ≥60 years, vaccination within the last 5 years was not associated with a reduced risk of all-cause CAP, but after exclusion of subjects who had received PPSV23 more than 5 years ago, vaccination was associated with a reduced risk for all-cause CAP hospitalization (25%; 2–42) [31]. In a case-control study carried out in three Spanish regions five years after the introduction of PPSV23, VE against CAP hospitalization was 23.6% (0.9–41) [27].

A meta-analysis of the last Cochrane review found a pooled estimate of vaccine efficacy of 28% (7–44) for all-cause pneumonia, but there was substantial variability in the effect estimate due to heterogeneity, and the effectiveness of vaccination in preventing all-cause pneumonia in adults could not be demonstrated [32]. In another meta-analysis, the pooled effect estimate for preventing CAP was 7% (-19 to 28) among individuals who were vaccinated in the previous five years [33].

In a large case-control study in Connecticut, vaccination was effective against invasive pneumococcal disease, but no VE was found in subjects aged ≥65 years vaccinated more than 5 years ago [34]. A retrospective case-control study in Israel in subjects aged ≥65 years found that PPSV23 administered in the 5 previous years was effective against invasive pneumococcal disease, but no protective effect against hospital-treated pneumonia was found (aOR: 1.01; 0.97–1.04) [35]. In Australia, using the screening method, VE against invasive pneumococcal disease in subjects aged ≥65 years in which those who received the vaccine in the previous 5 years was 71% (54–82) [36]. An indirect cohort study in Spain found that PPSV23 in the previous 5 years prevented 44% (24–60) of all invasive pneumococcal disease serotypes included in the vaccine [37]. The estimate of these authors was clearly higher than ours, but this seems logical because they investigated prevention against *S*. *pneumoniae* disease in which VE is expected to be higher than against all CAP.

A possible explanation for the differences found in PPSV23 effectiveness against CAP hospitalization might lie in the influence of the circulation of influenza viruses and other environmental factors on bacterial complications [30,38–41]. To avoid the possible influence of these factors, we defined the weeks when the influenza virus was circulating in Spain in each season and introduced this variable in the estimate of PPSV23 effectiveness; however, this was not done in the studies with negative results, making comparisons difficult.

No conclusions can be drawn on our estimates of VE in different age groups due to a lack of statistical power, as also suggested in the study by Vila-Córcoles et al. [42].

In the present study, the effectiveness of PPSV23 in preventing ICU admission or death was 28.1% (95% CI -14.3–56.9) for all patients and 40.9% when subjects vaccinated more than 5 years previously were excluded (S2 Table). In a Spanish cohort study, vaccination within the last 5 years was not associated with a reduced risk of death from CAP (1.04; 0.64–1.69) [31]. Other studies of subjects vaccinated at any time found that the protective effect of the PPSV23 against death was higher in immunocompetent than in immunocompromised patients [5,43,44]. However, although effectiveness is attenuated in immunocompromised patients, these are precisely the patients who have the most to gain by immunization as the risk of death is higher [45].

In a recent randomized, placebo-controlled trial, the VE of the PCV13 against CAP in the elderly was 45.6% (21.8–62.5) [46] and the benefit of the conjugate vaccine versus the polysaccharide vaccine in avoiding deaths has also been reported [47]. Therefore, it may be questioned whether the use of PCV13 would be more useful in preventing CAP in the elderly. The results of several impact studies suggest that routine immunization with pneumococcal conjugate vaccines in children reduces the incidence of disease due to conjugate vaccine serotypes in the elderly [48–51]. However, the epidemiology of specific serotypes evolves [52] and the duration of immunity and the need for revaccination is not currently clear [53]. On the other hand, the effect of herd immunity due to the direct effect of adult PCV13 vaccination remains unclear [54]. In fact, the CDC states that routine PCV13 vaccination in adults aged ≥65 years will be reevaluated in 2018 [10].

A cost-effectiveness study carried out in the UK show that the incidence of vaccine-type disease will probably be very low due to the wider benefits of childhood PCV13 vaccination and that a specific PCV13 vaccination program targeting the immunocompetent elderly would not be cost-effective [55]. A Dutch study found that PCV13 vaccination of immunocompetent persons aged 65–74 years was not cost-effective, although vaccination of high-risk individuals aged 65–74 years was cost-saving [56].

The results of the present and above-mentioned studies, together with the fact that PPSV23 includes eleven serotypes not found in PCV13, support the current indication for PPSV23 vaccination in the elderly and the interest in maintaining continuous surveillance of disease-causing serotypes in the elderly in order to evaluate the potential benefit and cost-effectiveness of expanding PCV13 vaccination to all elderly persons.

One limitation of the present study is that the main analysis was carried out considering persons not vaccinated in the previous five years as unvaccinated, which could have led to an underestimate of the VE. Another possible limitation is that interviewers knew whether interviewees were cases or controls, influencing information gathering. The same protocol was followed in cases and controls and information on the vaccination history was collected from information collected in medical records, vaccination cards or registers before the study began. Therefore, it is unlikely that the results were affected by this possible information bias.

Most potential confounding factors described in the literature, including influenza vaccination and comorbidities, were taken into account and their possible effect limited by adjustment [45]. Thus, although some residual confounding cannot be ruled out, this is unlikely to have invalidated the results.

Cases were older and had more medical risk conditions than controls, and therefore were more likely to receive the vaccine, but since a propensity score was used for the adjustment it seems unlikely that this would invalidate the results.

Finally, because the number of *Streptococcus pneumoniae* cases found was very limited, it was not possible to estimate VE in cases of *S*. *pneumoniae* CAP due to lack of statistical power.

In conclusion, the results of this study indicate that PPSV23 vaccination showed a modest trend to avoidance of hospitalization due to CAP in elderly subjects and in preventing death or ICU admission in elderly patients hospitalized with a diagnosis of CAP. The current indication for PPSV23 vaccination in the elderly should be maintained but continuous surveillance of disease-causing serotypes in this population is required.

## Supporting information

S1 Table Crude and adjusted effectiveness of PPSV23 against hospitalization due to community-acquired pneumonia. (DOCX)

S2 Table Effectiveness of the PPSV23 in avoiding intensive care unit admission or death in hospitalized patients with community-acquired pneumonia. (DOCX)
