# Supplementary material for: Effectiveness of 23-valent pneumococcal polysaccharide vaccination in preventing community-acquired pneumonia hospitalization and severe outcomes in the elderly in Spain
Source: PLoS One. 2017 Feb 10;12(2):e0171943. doi: 10.1371/journal.pone.0171943 (PMC5302444; doi:10.1371/journal.pone.0171943)
Supplement: S1 Table — (DOCX) [file pone.0171943.s001.docx]

**S1 Table**. **Crude and adjusted effectiveness of PPSV23 against hospitalization due to community-acquired pneumonia**

|  | Cases  vaccinated/N (%) | Controls  vaccinated/N (%) | Crude vaccine effectiveness^a^ (95% CI) | p-value | Adjusted vaccine effectiveness^a^ (95% CI) | p-value |
| --- | --- | --- | --- | --- | --- | --- |
| All | 259/1895 (13.7%) | 272/1895 (14.4%) | -5.7% (-35.2 – 17.4) | 0.66 | 6.1% (-21.8 – 27.6) | 0.64^b^ |
| 65-74 years | 116/592 (19.6%) | 133/592 (22.5%) | 7.7% (-31.1 – 35.0) | 0.65 | 13.2% (-26.0 – 40.0) | 0.46^c^ |
| 75-84 years | 94/879 (10.7%) | 95/879 (10.8%) | -13.2% (-75.1 – 26.9) | 0.58 | -2.9% (-62.6 – 34.9) | 0.90^d^ |
| ≥85 years | 49/424 (11.6%) | 44/424 (10.4%) | -35.0% (-140.7 – 24.3) | 0.31 | -16.4% (-116.7 – 37.5) | 0.63^e^ |

Adjusted for the propensity score. ^a^ Excluding cases and controls vaccinated more than 5 years previously. Statistical power: ^a^8%, ^b^14%, ^c^4%, ^d^8%
