# Supplementary material for: Effectiveness of 23-valent pneumococcal polysaccharide vaccination in preventing community-acquired pneumonia hospitalization and severe outcomes in the elderly in Spain
Source: PLoS One. 2017 Feb 10;12(2):e0171943. doi: 10.1371/journal.pone.0171943 (PMC5302444; doi:10.1371/journal.pone.0171943)
Supplement: S2 Table — (DOCX) [file pone.0171943.s002.docx]

**S2 Table**. **Effectiveness of the PPSV23 in avoiding intensive care unit admission or death in hospitalized patients with community-acquired pneumonia**

|  | Severe outcomes vaccinated/N (%) | Non severe outcomes vaccinated/N (%) | Crude vaccine effectiveness^a^ (95% CI) | p-value | Adjusted vaccine effectiveness^a^ (95% CI) | p-value |
| --- | --- | --- | --- | --- | --- | --- |
| All | 21/149 (14.1%) | 238/1032 (23.1%) | 45.3% (13.1 – 67.1) | 0.01 | 40.9% (2.9 – 65.6) | 0.04 |
| Immunocompetent | 10/73 (13.7%) | 147/626 (23.5%) | 48.3% (1.0 – 75.6) | 0.05 | 47.1% (-5.8 – 75.9) | 0.09^b^ |
| Immunocompromised | 11/76 (14.5%) | 91/406 (22.4%) | 41.4% (-11.5 – 71.8) | 0.12 | 35.6% (-27.8 – 70.1) | 0.23^c^ |

Adjusted for the propensity score. ^a^ Excluding cases and controls vaccinated more than 5 years previously. Statistical power: ^b^45%, ^c^26%
